# PP108 A Systematic Review Of Reactogenicity And Safety Of Recombinant Zoster Vaccine For Prevention Of Herpes Zoster In Adults

**DOI:** 10.1017/S0266462324002691

**Published:** 2025-01-07

**Authors:** Emma Reece, Orla Jenkins, Aoife Bergin, Ellen Reidy, David Byrne, Carol Mc Loughlin, Joan Quigley, Conor Teljeur, Patricia Harrington, Máirín Ryan

## Abstract

**Introduction:**

Herpes zoster (HZ), also known as shingles, is characterized by a vesicular skin rash, often associated with acute pain and itching. The safety profile of the recombinant zoster vaccine (RZV) in adults aged 50 years and older and in adults aged 18 and older who are at increased risk of HZ was assessed in this systematic review.

**Methods:**

A comprehensive electronic search was performed in Embase, MEDLINE, the Cochrane Library, and clinical trials registries. Searches were limited to the period from 2008 to July 2023. Article screening and data extraction were carried out by two independent reviewers. Risk of bias was assessed using the Cochrane revised Risk of Bias 2 (RoB2) tool for randomized controlled trials (RCTs). The Risk of Bias in Non-Randomized Studies of Interventions (ROBINS-I) tool was used to assess the quality of non-randomized studies. An adapted version of the Newcastle–Ottawa Scale was used for the appraisal of quality of non-comparative studies.

**Results:**

Eighteen RCTs, four observational cohort studies, seven single-arm trials, and 11 single-arm observational studies were identified. Compared with placebo, solicited local (RZV: 74.1 to 84.0%; placebo: 7.9 to 11.9%) and systemic reactions (RZV: 53.0 to 66.1%; placebo: 6 to 11.4%) were more common in the vaccinated cohorts. Reactions were generally transient and mild to moderate in intensity. The most frequent reactions reported were pain at the reaction site, fatigue, and myalgia. The incidence of potential immune-mediated diseases (pIMDS), serious adverse events (SAEs), and fatalities was similar in vaccine and placebo groups. No SAEs, pIMDs, or deaths were reported as vaccine related.

**Conclusions:**

The available data on RZV shows that while local and systemic adverse events are common with RZV, these are typically transient, and SAEs are uncommon in both the general population and those at increased risk of HZ.

